# Challenges experienced by nurse educators developing postgraduate nursing diploma curriculum programmes, Gauteng

**DOI:** 10.4102/curationis.v46i1.2447

**Published:** 2023-08-29

**Authors:** Nokuthula D. Buthelezi, Khumoetsile D. Shopo

**Affiliations:** 1 Quality in Nursing and Midwifery (NuMIQ), Faculty of Health Sciences, North-West University, Potchefstroom, South Africa; 2 Gauteng Department of Health, Johannesburg, South Africa

**Keywords:** competency-based approach, curriculum, experience, nurse educator, transition

## Abstract

**Background:**

Nursing education’s positioning within higher education mandated public nursing education institutions to develop competent nurses to manage diverse disease profiles of the country. Nurse educators were tasked to develop a competency-based curriculum with emphasis on primary healthcare to help prepare nurses to be independent, leaders, researchers, and critical thinkers.

**Objectives:**

To explore and describe the challenges experienced by nurse educators in Gauteng when developing the curriculum for the postgraduate nursing diploma programmes.

**Method:**

An exploratory descriptive qualitative research design was used. Purposive sampling was followed to select the participants based on the inclusion criteria. Four focus group interviews were conducted, comprising of six participants each, leading to a sample of 30. Data collection were between March 2022 and April 2022. Thematic data analysis were performed following Tesch’s eight steps of analysis.

**Results:**

Themes that emerged during data analysis were psychological and emotional impact, challenges with communication and interpersonal relations, nurse educators experienced transformation and empowerment, nurse educators encountered barriers that impacted on their allocated tasks, and, nurse educators demonstrated resilience with the curriculum development processes.

**Conclusion:**

Participants reported positive and negative experiences they encountered during curriculum development. The findings revealed that nurse educators need support when involved in curriculum development, for instance, managerial, administrative, technological, financial, and most importantly capacitation, as this could enable them to work effectively without deterrents.

**Contribution:**

This study highlights the need to train and support nurse educators when developing a curriculum to equip them with the necessary competencies.

## Introduction

The Department of Health (2013:4, 8) identified the need to produce competent nurses to manage the country’s burden of disease and meet South Africa’s healthcare needs. Following a National Nursing Summit held in 2011, a nursing education, training and practice strategy was developed. The summit recommended that nursing education should be positioned within the higher education system. Blaauw, Ditlopo and Rispel (2014:1) concluded that a change in legislation was required for the positioning of nursing education qualifications in higher education. Restructuring the nursing curriculum from being content based to outcome based and a shift of focus to primary healthcare would ensure the development and production of graduates with a comprehensive knowledge base, who can undertake practice-related research and competence in an evidence-based practice (Mtshali & Zwane 2019:2).

Planning transformation of nursing education involved various stakeholders, including the statutory body for nursing, various nursing and education organisations, the private sector and health sector unions (Nyoni 2020:3). The regulations for postgraduate diploma (PG Dip) programmes were promulgated by the Minister of Health in terms of the *Nursing Act* of 2005. The promulgation was performed after consultation with the South African Nursing Council (SANC) as the regulatory body for Nursing in the country.

The regulations for PG Dip programmes and curriculum frameworks were developed to enable accredited or prospective Nursing Education Institutions (NEIs) to develop programmes that would prepare competent and safe nurse practitioners in line with competencies for specialisation (SANC 2020:8). The curriculum development process at the NEIs commenced soon after the publication of the regulations for the PG Dip programmes in mid-2020.

Murdoch-Eaton, Louw and Bezuidenhout (2016:15) concurred that nurse training should be strengthened, and this can be achieved through constant revision, implementation and evaluation of the curriculum to help produce competent and dedicated health professionals who will be able to respond to the diverse population’s needs. Botma (2014:1876–1891) reported in a study conducted in Lesotho, a small sub-Saharan African country, that a team of nurse educators involved in the curriculum development process described it as confusing and exhausting, while others described the process as the most difficult task they had ever undertaken. Keating and DeBoor (eds. 2018:32) asserted that NEIs should not assume all nurse educators are familiar with curriculum design and development; therefore orientation is vital before the commencement of the process. Nurse educators in training should receive in-depth knowledge in teaching and learning, evaluation and assessment, curriculum development and the nurse educator’s role (Booth et al. 2016:54).

The first author, a nurse educator at a public NEI in Gauteng, observed that nurse educators were overwhelmed, having to adapt to changes in curriculum design and development using evidence-based guidelines and policies. The objective of this study was to explore and describe the challenges experienced by nurse educators when engaged in curriculum development for the new PG Dip programmes. From this background, the research question was: ‘What are the challenges that nurse educators experienced when developing a curriculum for the new postgraduate diploma programmes?’

## Research methods and design

### Study design

An exploratory descriptive qualitative research design (Gray, Grove & Sutherland 2017:71) enabled the authors to explore the challenges that nurse educators experienced during the process of developing the curriculum for the PG Dips. Colorafi and Evans (2016:17) described a qualitative descriptive research design method as a comprehensive summary of events experienced by individuals or groups. Gray et al. (2017:70) affirmed that using a qualitative exploratory approach enables the authors to explore and describe the life experiences of the participants.

### Setting

The setting was four campuses of a public NEI in Gauteng that will be offering the PG Dip programmes after accreditation. The NEI is a government institution with the main office in Johannesburg, Central Business District (CBD).

### Study population and sampling strategy

The study’s target population was nurse educators from four campuses of the NEI, who will be involved in the teaching and learning of the PG Dip programmes after accreditation. Participants were selected using a non-probability all-inclusive purposive sampling technique (Nieswiadomy & Bailey 2018:379) because it enabled the participants to provide information the authors sought about what they had experienced during the curriculum development process (Nieswiadomy & Bailey 2018:177). Thirty nurse educators, meeting the inclusion criteria, participated. Inclusion criteria comprised of registered nurses with a postgraduate qualification in nursing education, registered with SANC and had been actively involved in the process of curriculum development.

### Data collection

Data collection was done through focus group interviews (FGIs) at each campus between March 2022 and April 2022. The FGIs allowed the authors to acquire a variety of responses from the participants (Barret & Twycross 2018:63). The duration of FGIs was 60–90 min. Interviews took place in the campus classrooms and boardroom.

Barret and Twycross (2018:63) described an FGI as a method of data collection in which an interviewer speaks to a group of 6 to 10 participants about issues related to the research question. An information session was conducted for prospective participants before giving consent to participate in the study. Data were collected by two moderators, with the first author sitting in as an observer because of her senior position; this was to allow the participants an opportunity to respond freely and openly to the questions asked. Interviews were audio recorded with permission from the participants, and field notes were written during the interviews to enrich the data collected. The COVID-19 restrictions were observed throughout data collection, for instance, social distancing, wearing of facial masks and availability of sanitisers and good ventilation. The following questions were asked during FGIs:

- Can you explain the impact the process of developing the new PG Dip programmes had on you as a nurse educator?
- What were the facilitating factors that assisted in developing the curriculum for the new PG Dip programmes?
- What were the barriers faced during the curriculum development process for the new PG Dip programmes?
- Can you please share how you overcame the barriers during the curriculum development process for the new PG Dip programmes?

The moderators used probing, clarifications, paraphrasing and summarising to obtain more clarity and in-depth information from participants.

### Data analysis

A professional transcriber transcribed the interviews verbatim. The authors used thematic analysis of data following Tesch’s eight steps for qualitative research (Creswell & Creswell 2018:291). Co-coding was performed by an independent coder with expertise in qualitative data analysis. This was conducted simultaneously with data collection. After the co-coder’s analysis, there was a consensus meeting to go through the findings to authenticate the data analysed. Analysed data, themes and sub-themes were further discussed with the supervisor to enhance credibility.

### Trustworthiness

To ensure trustworthiness, the authors should demonstrate credibility, transferability, dependability and confirmability (Polit & Beck 2017:559–560). The authors applied credibility by prolonged engagement during data collection, as well as persistent observation. Data were analysed by the first author and independent co-coder who is a qualitative researcher to ensure dependability. Credibility was ensured by member checking, prolonged engagement and documenting data accurately by verbatim transcription. By providing a thick description of the methodology, the authors ensured that other researchers will be able to replicate research in other settings. The authors, therefore, applied the transferability principle. Confirmability was ensured by the authors being truthful in the reporting of the research process, including results.

### Ethical considerations

The authors obtained approval from the Independent Review Board of a university before commencing the research; a reference number will be provided. The National Health Research Database (NHRD), the Acting Director of Research and Compliance and the Nursing Education Institution (NEI) Principal granted permission to conduct the research study at the NEI. The Campus Heads took the role of gatekeepers. Participants had the freedom to decide whether they wanted to participate in the study, thus maintaining the ethical principle of autonomy. Participants did not use their names during the collection of data as each received a number from 1 to 30, thus adhering to autonomy and confidentiality. By following the principle of justice, the participants’ selection was purposive and fair, according to the inclusion criteria. There were minimal risks because participants had no exposure to harm as per the principle of beneficence.

## Results

The findings of the study indicated the objective was met, which was to explore and describe challenges nurse educators experienced during curriculum development for the PG Dip programmes. Data collected from the FGIs generated 5 themes and 14 sub-themes. Participants’ socio-demographic profile is outlined in the next section.

### Socio-demographic profile

The participants (*n* = 30) were all women (100%), with a mean age of 58 years. In terms of ethnic background, the majority were African (97%), with one mixed race participant (3%). All participants had a bachelor’s degree qualification, majoring in Nursing Administration and Education, 16 had a Master’s degree in Nursing, one had a Master’s degree in Business Administration, 13 were studying towards a Master’s degree in Nursing, one participant was not pursuing a Master’s degree and four were studying towards a doctoral degree.

Table 1 outlines the themes and sub-themes as they emerged.

**TABLE 1:** Themes and subthemes from the study.

Themes	Sub-themes
**Theme 1**: Psychological and emotional impact	**Sub-theme 1.1**: Feelings of frustration and confusion **Sub-theme 1.2**: Feelings of helplessness and stress **Sub-theme 1.3**: Experiencing exhaustion and ill-health
**Theme 2**: Challenges with communication and interpersonal relations	**Sub-theme 2.1**: Alack of information affected the nurse educators’ contribution to the curriculum development **Sub-theme 2.2**: Poor communication culminated in reluctance to participate in the curriculum development activities **Sub-theme 2.3**: The need for belonging to the curriculum development team **Sub-theme 2.4:** Bullying from their colleagues
**Theme 3:** The nurse educators’ experience in transformation and empowerment	**Sub-theme 3.1:** Realisation of their strengths and weaknesses **Sub-theme 3.2:** Improved self-confidence through the process of learning how to develop a curriculum **Sub-theme 3.3:** Incorporation of facilitating factors in the curriculum development project
**Theme 4:** Barriers that nurse educators encountered that had an impact on their allocated curriculum development tasks	**Sub-theme 4.1:** A lack of tools of the trade and other supporting resources **Sub-theme 4.2**: Nurse educators supported the curriculum development project by using their own resources **Sub-theme 4.3**: A lack of support from the management
**Theme 5:** Nurse educators demonstrated resilience with the curriculum development processes	**Sub-theme 5.1:** Developing high levels of self-efficacy in dealing with the challenges

#### Theme 1: Psychological and emotional impact

Three sub-themes were generated from the first theme, psychological and emotional impact. Nurse educators who participated in the activities of the curriculum development reported feeling confused, frustrated, helpless, exhausted and unwell.

**Sub-theme 1.1: Feelings of frustration and confusion:** When nurse educators joined a team that had previously made headway with the initial stages of curriculum development, frustration and uncertainty became obvious. They were not instructed in the procedure or given feedback; instead, they were expected to proceed with the tasks of curriculum development without much support. This is demonstrated by the following verbatim quotes from the participants:

‘My experience was that of confusion and frustration as I was doing the … this … the programme alone especially at the beginning, without support from the very same campus I was working in. So, the support … I received it later, but at the beginning I was so frustrated and very confused, not knowing whether I’m right or wrong.’ (P13, FG2, Campus B)

‘My emotional experience regarding the PG Dips curriculum, for me emotionally it was quite frustrating and at the same time, it was, you know, there was a lot of mixed emotions with me. I was confused at some point.’ (P28, FG4, Campus C)

‘I was frustrated by the fact that I was never developed at all, personally, before I engaged in it, I had to actually develop a curriculum of a programme in 13 days. And I want to believe that is practically impossible, because I knew nothing about curriculum development, except the terminology having heard and learned about it when I was doing the BCur many years ago.’ (P2, FG1, Campus C)

Because they were to develop a curriculum without the appropriate guidance and support, nurse educators found the task frustrating and confusing. As this change introduced new and unknown components of the content to be taught, they might have been concerned about losing control of some areas of the curriculum (Keating 2015:56).

This study confirms that of Baron (2017:284), which concluded that when curriculum changes are implemented, the outcomes may not be as anticipated because certain nurse educators may be resistant to the change and express emotions of frustration.

**Sub-theme 1.2: Feelings of helplessness and stress:** Some participants experienced high levels of anxiety as the curriculum-development process moved along because they lacked guidance on how it should proceed. Some of the participants ultimately learned on their own:

‘Er … I felt … err … very overwhelmed and helpless at times, more especially … err … because with … you know, I … we … I felt that there was no direction. There was no direction and at times when you felt like you did not understand what you were expected to do, you didn’t even know whom to go to or to consult then. Therefore, really, it was such an overwhelming experience.’ (P21, FG3, Campus D)

‘During that … err … process, I felt so overwhelmed and very confused every day because there was no forwardness [*progress*]. Every time we’re like, in one place, because no one was leading us. No one had a clue. We felt all of us so drained, every day you felt so drained and it was a very, very hectic experience. Thank you.’ (P19, FG3, Campus D)

‘We did not know where to start. I didn’t want to hear the word “curriculum.” If we had students, I would prefer to be with them throughout. It was a case of the blind leading the blind.’ (P1, FG1, Campus C)

According to responses, the nurse educators’ participation in curriculum development affected their well-being. Some reported that because they put in a lot of overtime and did not get enough rest, they now get insomnia regularly. Some individuals believe that their level of stress played a role in the onset of hypertension.

**Sub-theme 1.3: Experiencing exhaustion and ill-health:** The well-being of nurse educators was reportedly impacted by their engagement in curriculum development. Some claimed that because they worked long hours and did not get enough sleep during the process, they now frequently get insomnia. Some have said their level of stress contributed to the development of hypertension:

‘Impacted negatively on my health, I developed hypertension. The experience was daunting. There were occasions when I did not sleep at all for two days.’ (P2, FG1, Campus C)

Some nurse educators associated their ill health with the process of developing the curriculum:

‘I was called back from my studies in September 2020 to assist with the curriculum. The pressure was so high. We were forced to go and study, now it’s 50/50 study leave. The pressure was so high I developed post-traumatic disorder.’ (P4, FG1, Campus C)

‘… so we were the only people who were expected to be driving, and driving is emotionally draining, physically and emotionally as well, … but … err … it was emotionally and physically draining, having to drive alternate weeks and not changing venue, being us who were to be exposed to rainy … rains … whatever, and busy roads. It was emotionally frustrating ….’ (P17, FG2, Campus B)

The curriculum development process, according to nurse educators, had an impact on their health. They had to work long hours and on weekends, which left them exhausted. Some of them were also held responsible for their health problems on the pressure of the timelines they were given to complete the tasks assigned to them for creating documents for their programmes. These results corroborate another study, which found that curriculum development was challenging and overwhelming for participants, who felt weary afterwards (Botma 2014:1891).

#### Theme 2: Challenges with communication and interpersonal relations

According to nurse educators, the information provided to them to help with curriculum development was insufficient, which in turn had an impact on their contribution. They added that as the curriculum development process advanced, they realised there was a lack of communication, which led to a reluctance to take part in activities and a need to be a member of the curriculum development team.

**Sub-theme 2.1: A lack of information affected the nurse educators’ contribution to curriculum development:** Before starting to build a curriculum, nurse educators asserted they did not get any information. They found it difficult to understand what was expected of them in terms of input because of a lack of information:

‘And the other barrier would be … I think lack of information or lack of in-service on my part, because with … uhh … sufficient information I would have been able to master the curriculum development successfully.’ (P28, FG4, Campus A)

‘I had no clue of what was happening everything was Greek to me. No book to follow. There was nothing written down. The process was not explained, everybody had her own views.’ (P4, FG1, Campus C)

Some participants indicated that they were unsure of what was expected of them because they did not understand how the curriculum was developed. This conclusion is reinforced by research by Baron (2017:282), who observed that because of a lack of understanding of curriculum development, nurse educators initially believed they needed to change the outdated curriculum but subsequently realised they needed to completely redesign it. He further asserted that nurse educators needed instruction on the procedure to be followed before realising that all that was needed from them was coordination and time to develop the curriculum (Baron 2017:282).

One participant reported that:

‘… when we meet with other campuses, we are the people who are lagging behind and there are things that we don’t know, people are ahead of us and I think it’s a lack of information sharing and lack of feedback, and it was not been addressed.’ (P15, FG2, Campus B)

The lack of effective communication about the schedule for developing the curriculum on campus was cited by some nurse educators; in some cases, they were compelled to travel right away to another campus and join the curriculum team without any warning or preparation.

**Sub-theme 2.2: Poor communication culminated in a reluctance to participate in the curriculum development activities:** Some nurse educators emphasised the fact that there was poor communication on campus regarding the schedule for curriculum development; occasionally, they were required to go immediately to another campus and join the curriculum team without warning or preparation:

‘I remember there was an incident where I thought … “you know what, maybe I don’t belong here” I even wrote a resignation letter because they were telling me at 11:00 that “you’re supposed to be at another campus by 12 noon, the meeting is starting, why are you still here?” and I was never communicated about that.’ (P12, FG2, Campus B)

According to research by Iwasiw, Andrusyszyn and Goldenberg (2020), excellent communication during the curriculum development process from top management down to nurse educators helps to produce a well-developed and powerful curriculum. This is because all parties can seek and receive advice or feedback from one another, which could facilitate the project’s progress and efficiency. The results of this study demonstrate the importance of managers’ excellent communication in ensuring that nurse educators are at ease and aware of what is expected of them during the curriculum creation process.

**Sub-theme 2.3: The need for belonging to the curriculum development team:** Some participants reported that they occasionally felt alienated; they did not completely engage in the process because they were denied an opportunity at their campuses for a certain time. One nurse educator would be assigned to join the team 1 day and the next day another nurse educator would be assigned. It was challenging for them because just as they believed they had a clear understanding of the process, they received news that another person would be joining the team. Consequently, this had a negative impact on following up on the process. This eventually made them feel not being part thereof:

‘You’ll go there being blank, when you arrive there, there’s no one who’ll … who’s sitting there to fill you in, like you’re just there because you needed to be there just to say I’m present, I’m there and at the end of the day it made me feel as if I’m just useless or hopeless thing that … to be tossed around and to be told what to do, when to do, whenever the superior feel like that because there was no enough communication, according to me … uhm … how they were approaching us as well, it wasn’t … like tomorrow you’re going where-where, it was right now.’ (P12, FG2, Campus B)

‘So, it was … I felt like we were being rated inferior or what I don’t know. And it’s like … like I said earlier, to say we’re just being called for the sake of being called or for the sake of the fact that we belong to the NEI but not that our … err … err … our inputs were of that value.’ (P17, FG2, Campus B).

When their managers just directed them to join the curriculum development team without providing any further information, some nurse educators on campuses felt alienated by them.

The curriculum development team needed strong leadership support throughout this process to be motivated and encouraged; ongoing support boosts employee morale. According to Botma (2014:1891), the NEI should exhibit the quality of a curriculum that would draw candidates to the profession. This can only be achieved with the assistance of senior management and team members.

**Sub-theme 2.4: Bullying from their colleagues:** The relationship between nurse educators was compromised because some felt overwhelmed and subjected to bullying during curriculum creation:

‘The impact was bad, I met bullies. Now I’m scared of some colleagues. I was anxious. Personal relations were affected.’ (P3, FG1, Campus C)

‘We do have age differences and others are more vocal than others and when you become vocal others start using their age and say you’re just a child do what I’m telling you to do. It shouldn’t mean that me having a small stature I must be bullied in a way kind of.’ (P7, FG1, Campus C)

Bullying is a critical issue in the workplace, and this study shows that bullying does occur in higher education institutions. In addition, Chiang, Chapman and Elder (2011:5) claimed that including nurse educators in the curriculum development process could lead to different viewpoints about what should be included in the curriculum, which could lead to disagreement and potential bullying of some lecturers. In this study, the novice nurse educators reported on having had arguments with senior nurse educators, who in turn allegedly persecuted them.

#### Theme 3: The nurse educators’ experience in transformation and empowerment

The development of the curriculum, according to nurse educators, helped them to identify their strengths and weaknesses. More importantly, the project increased their self-confidence as they learned how to develop a curriculum by utilising several enabling variables.

**Sub-theme 3.1: Realisation of their strengths and weaknesses:** Some nurse educators acknowledged they had learned a lot during the curriculum-development process and were willing to take part again if necessary:

‘One, I realised my strength and the weakness and I get to learn the macro, the me … micro. Meso and the micro and the … I get to understand them very well and clearly and how the ELO’s [*exit level outcomes*] and all those terminologies come about and their explanations, so I can say now if they can say “go for curriculum and start the programme from the beginning,” I can do that within two months by now.’ (P12, FG2, Campus B)

‘I am grateful to say that ehm … the way I am now I understand the principles, I don’t know them 100% but then I can confidently say one may give me a new curriculum I’ll know what to do and I’ll … [*pause*]. Yes, I am maybe 70% confident though not 100%, but it’s a trial and error type of thing but my knowledge shift is err … very positive and I’m confident that I can start the curriculum from afresh.’ (P9, FG1, Campus C)

‘Uhm … I have also learned a lot from … from … from … from participating in that curriculum development and one thing that’s done … that stands out for me is the … is the chance that I had.’ (P16, FG2, Campus B)

According to Balcom, Kuhnke and Roy (2021:9), nurse educators’ involvement in curriculum development assists them in recognising their sense of self and self-identities because their knowledge significantly increased. Albilehi, Han and Desmidt (2013:194) also concurred that nurse educators’ participation in curriculum development helped them to improve their attitudes towards the changes in curriculum approach because they learnt, thought about and engaged in activities that shaped the entire curriculum. After the procedure, they felt assured in their skill and knowledge of the subject matter they will be teaching (Albilehi et al. 2013:194).

**Sub-theme 3.2: Improved self-confidence through the process of learning how to develop a curriculum:** At the beginning of the curriculum development process, nurse educators claimed to have faced challenges, but as days progressed, they read and searched for information and through discussions with other colleagues, their confidence grew. At the conclusion of the process, the majority felt more confident about developing a curriculum:

‘Uhm … I learned a lot about curriculum development, from the beginning until the end, and I’m kind of thinking now I can … I’m a master, I can do any curriculum development of any programme because I learned on my own. I’m a master of that. I’m very thankful for having that experience, Ma’am.’ (P22, FG3, Campus D).

‘So, I’ve learned a lot to an extent that where I am now, I’m the person that is even guiding them. So, I have learned so much, really, one would think that it was a waste of time having to do it without the regulation but … err … it paid off well after the regulation was finally given out by the Nursing Council but I have learned a lot.’ (P17, FG2, Campus B).

‘As I’m going to be teaching the very programme, I will be understanding how we arrived at certain decisions so I think that is going to make me a better educator in terms of the programme that I will be teaching just because I have participated in the programme and a lot of things were clarified ….’ (P16, FG2, Campus B).

**Sub-theme 3.3: Incorporation of facilitating factors in the curriculum development project:** According to nurse educators, adding several facilitating variables made developing the curriculum easier for them. The key facilitating aspects mentioned are teamwork, mutual support and senior educators. Some claimed that there were no facilitating factors to help them in this process as they felt unprepared before the process commenced, which prevented them from participating:

‘Lack of knowledge brought us together. We were all empty [*laughter by participants*]. We all fumbled together; this motivated me.’ (P1, FG1, Campus C)

‘The teamwork that we had made things easier because we were able to discuss, you know, go through whatever documents we had, go through whatever problem we had and then we’d ask the Seniors or those who were there for curriculum development for advice and then they’ll show us how to do it.’ (P30, FG4, Campus C)

‘Teamwork, support from my seniors [*lecturers*]. I want to thank you because you really helped me.’ (P6, FG1, Campus C).

‘The breakaway teams where we had to go to hotels, that also helped a lot because we’d work from morning to evening without a break and there was much more progress that we saw … err … coming up.’ (P26, FG4, Campus C)

The nurse educators’ participation in curriculum development was made possible through effective cooperation and peer support. Other nurse educators offered support to other members who were feeling overwhelmed and frustrated. They were able to acquire clarity through the assistance provided by the curriculum champions, which further facilitated the process. Iwasiw et al. (2020:25) state that obtaining support from peers or other sources, whether it be financial, administrative, technological or administrative support, could be beneficial to the team because it lessens the frustration and stress that they are more likely to experience during the curriculum development process.

#### Theme 4: Barriers that nurse educators encountered that had an impact on their allocated curriculum development tasks

During the development of the curriculum, nurse educators identified several obstacles, including a shortage of phones, connectivity, assistance and funding. The lack of financial support for nurse educators during the curriculum development process, they stated, felt like neglect given that other programmes did receive support.

**Sub-theme 4.1: A lack of tools of the trade and other supporting resources:** Concerns about the lack of resources needed for usage during curriculum development were raised by nurse educators. According to them, there was no training provided before they started the process, which made it difficult for them to get on board. They also complained about a lack of rules for PG Dip programmes, a lack of funding, problems with certain laptops while others were broken and connectivity issues as nurse educators did not have modems, which stopped them from accessing the internet:

‘Err … thank you, Ma’am! Err … with the addition to what Ma’am has just said, the other barrier on my side was the issue of the laptop, our laptops that were non-functional, yet we were expected to work with them.’ (P12, FG2, Campus B)

‘… lack of telephones, whereby some information, you would just notify your colleagues about the situation that you were currently in, lack of telephones. We had to use our own cell phones and we were not being given even … err … err … money for airtime or even for data.’ (P17, FG2, Campus B)

‘… in our path as mental health practitioners in our team … uhh … allocation of funds related to … uhm … processes like people having something to eat, people going to hotels for two weeks. We were not allocated anything; it was based on a zero-budget compared to others.’ (P22, FG3, Campus D)

In addition to the lack of telephones, which made it impossible for them to consult or contact colleagues at other campuses, nurse educators also mentioned that there were no published rules and that the process of publishing them took a long time. This result is similar to that of Dillard and Siktberg (2013:87), who identified a resource shortage as one of the obstacles that would prevent nurse educators from supporting curricular change.

**Sub-theme 4.2: Nurse educators supported the curriculum development project by using their own resources:** To prevent delays, nurse educators found themselves having to use their own resources. They promoted progress by utilising their own data and phones:

‘… And with regard to the internet interruptions and all that, specifically referring to the Wi-fi, I had to just improvise and buy my own data so that I’m not interrupted in the interim.’ (P28, FG4, Campus C)

‘… I had to use my own money to buy airtime so that I could communicate with my colleagues from the other institution and otherwise, apart from that, I really cannot say anything.’ (P17, FG2, Campus B)

‘We had to use our own cell phones and we were not being given even … err … err … money for airtime or even for data.’ (P17, FG2, Campus B)

Developing a curriculum with limited resources tends to hinder the process. As cited by Iwasiw et al. (2020:94) that without tangible institutional resources, curriculum work cannot proceed successfully. It was then up to the nurse educators to decide on using their own resources to foster progress.

**Sub-theme 4.3: A lack of support from the management:** As shown in their exact statements below, nurse educators asserted that they did not receive adequate support from management at campuses during the process of developing a curriculum for the PG Dips. The main obstacle was this barrier, which affected all campuses. However, because the team members were supportive of one another, they were encouraged to move forward with the project. The management overlooked the appeals for support from nurse educators. Others became frustrated as a result because they were unsure of what to do or whom to turn to:

‘The first barrier that is still a thorn in the flesh, is … err … management support from other campuses, whereby you find that … uhm … they didn’t want to release the participant to come over so that we could work on curriculum development together and we … we really … we’ve ended up having different people coming, and now we have to like, go in-depth and updating them on the process and the progress, and that became a barrier … uhm … management support hindering the progress of the … of this particular curriculum.’ (P29, FG4, Campus C)

‘Err … I think the second barrier it’s … err … the lack of support, you know. Err … Because sometimes when you’re stuck, you didn’t even know who to go to … err … who to consult. So, I feel … err … lack of support also contri … err … is also a barrier … was also a barrier. Thank you.’ (P23, FG3, Campus D).

When a new curriculum is being developed, nurse educators go through a paradigm shift as they have to transition from the familiar to the unknown, which they may view as a monster (Botma 2014:1891). The repercussions of not receiving support were detrimental to the nurse educators, who could have easily lost interest and morale as a result:

‘I want to extend to say when I needed support and asking for support, because I was feeling frustrated, I … we were in a meeting with our big bosses. I asked for support for the team that I was leading. They told me: “you can read and write.”’ (P22, FG3, Campus D)

The research findings on this theme are consistent with those of other authors. In a study on teachers’ participation in curriculum development, Alsubaie (2016:106) found conclusive evidence that the empowerment of educators through training and workshops is possible. The author further attested to the success of curriculum development and implementation by stating that giving educators the information and skills in curriculum development is essential. Keating (2015:169) argued that all departments must support the curriculum development process in NEIs because the team will undoubtedly use all available tools to get the project completed.

A lack of resources could impede the development of a curriculum because they are needed for administrative, library, technological, teaching and learning system support, among other things (Keating 2015:169–172). Being mentored by more experienced nurse educators while developing a curriculum is viewed as a form of assistance as it allows novice nurse educators to observe discussions, deliberations, ideas and debates that may be beneficial to them (Keating 2015:54).

#### Theme 5: Nurse educators demonstrated resilience with the curriculum development processes

This finding illustrates how resilient nurse educators were in the midst of the difficult challenges they faced while developing the curriculum. The theme has one sub-theme discussed below.

**Sub-theme 5.1: Developing high levels of self-efficacy in dealing with the challenges experienced:** According to nurse educators, to succeed with the challenges presented, they had to take matters into their own hands. They accepted the daily drive to the designated locations, some put in extra effort to fulfil tasks and information sharing was done. Other participants suspended their studies to concentrate on the curriculum development process, as reported below:

‘Okay … I overcame … we overcame the challenges of travelling, arriving home late, sleeping late, and going to class. You’re expected to do what you’re supposed to do, we just made peace with everything. And we accepted that.’ (P18, FG2, Campus B)

‘Uhm … To make sure that the team succeeded in mitigating the challenges, some of us had to work overnight. Uhm … You worked overnight so that you made sure the team succeeds. And for the low morale and the low motivation, we were motivating ourselves as a team to say, “Let’s do it!” …. Let’s complete it because you are the black sheep of the family. Just continue doing it.’ (P22, FG3, Campus D).

‘We were reading like we were doing our PhDs. [*background laughter*] We had to share information, we consulted, we were searching the internet, looking, trying to understand, you know, the … the … the … the contents of the various documents and unfortunately some of them we were actually … not actually aware of them, but we read, we even had to abandon our own studies. Some of us we could not even study, Ma’am, because of the very fact that we had to focus on this, uhm, the curriculum development neh.’ (P21, FG3, Campus D).

The self-efficacy of nurse educators can be interpreted as both a cause and an outcome of transformative learning processes (Balcom et al. 2021:10). In their study, Iwasiw et al. (2020:36) observed that the learning that transpired during this whole process became beneficial, and as a result, it strengthened their knowledge and competence in developing a curriculum, which in turn improved their self-efficacy and, ultimately, the quality of the curriculum they generated. This study confirms their results. In addition, Balcom et al. (2021:7) acknowledged that developing a curriculum for the new programmes helped nurse educators to develop a substantial sense of resilience and commitment to work diligently on developing the curriculum and saw it through to submission to SANC and Council on Higher Education (CHE) for accreditation.

## Discussion

The participants reported on a variety of challenges they faced when developing the curricula for the PG Dip programmes. The study’s objective of exploring and describing the challenges Gauteng nurse educators encountered in developing the curriculum for the new PG Dips programmes was therefore achieved. Only a minimal amount of information was obtained when the authors searched for data to support the topic. Participants reported feeling frustrated, confused, helpless, stressed and in some cases, ill health occurred as a result of their lack of preparation for the task and inadequate guidance. This result is comparable to that of Balcom et al. (2021:7), who suggested that the change from a familiar curriculum to one they had never encountered before made nurse educators feel concerned. They were unfamiliar with the process of developing a curriculum from scratch for programmes that adhered to the higher education quality sub-framework.

According to Keating (2015:56), people who are involved in a change process may experience a range of emotions, especially if they are unsure of where to begin and how to proceed, which can lead to frustration and confusion. They discovered that they had to work on weekends and for long hours, and their families suffered as a result.

The main issue mentioned by the participants was that before the process began, they did not have the capacity to develop a curriculum. They found it difficult to understand what to do next because of this. When the initial process of developing the curriculum for the PG Dips began in July 2018, SANC had not yet promulgated the regulations or published guidelines for the PG Dips, which added to the challenge. Some disagreements that broke out during discussions resulted in the bullying of novice nurse educators by their seniors. According to Iwasiw and Goldenberg (2014:8) team members may debate and argue during discussions, which can result in conflicts.

Because of learning and discovering new material, nurse educators reported an increase in confidence as they developed the curriculum. Some even described the early phases of the process as a roller coaster event, but they overcame it with perseverance and peer support. The help provided to the postgraduate curriculum development team by a curriculum champion helped nurse educators gain more information about curriculum development, and as they gradually came to comprehend the procedures involved, they started to feel more confident in themselves. In order to improve their morale, Baron (2017:285) contends that supporting educators during the process of curriculum reform or change is essential.

A lack of necessary tools was one of the key obstacles, some had no laptops and those who did have, experienced technical glitches. Accessing the internet was a challenge as connectivity at some campuses was at times poor while others had no Wi-Fi. Billings and Halstead (2019:87) attest that a lack of resources is a barrier when undertaking curriculum development, as this may result in resistance from educators to continue participating in this process.

They overcame some of the barriers by using their own laptops and data to access the internet. It was only after the intervention of senior management that they received Wi-Fi routers, and it enabled nurse educators to work with ease. Their ability to work as a team and be resilient helped them overcome their obstacles. According to Billings and Halstead (2019:312), transforming a curriculum may be challenging because nurse educators may feel as if they no longer have control over it. Resistance may emerge from this, which might make the process challenging. They proceed to assert that there may be mechanisms put in place to support the transitions and aid nurse educators in becoming familiar with the new curriculum.

Self-efficacy is a result of an individual’s self-belief in their ability to complete a task (Bandura 1986). Thus, great self-efficacy and tenacity will probably result in improved performance and productivity (Cherian & Jacob 2013:80). Nurse educators became committed to completing the assignment and learning. They persevered by supporting and advising each other, and they even used their own resources to make sure there was no delay in the process when challenges arose during the process. Nurse educators had to work long hours, read documents related to curriculum development and conduct information processing searches.

### Strengths and limitations

The study gave nurse educators a chance to discuss their perceptions of the curriculum development process. They were able to reflect on both the positive and negative impacts this process had on their personal and professional life as they reported on it. At four NEI campuses, focus groups with 6 to 10 nurse educators each were conducted to collect data. The first author was only able to recruit one nurse educator from Campus A to participate, posing a constraint. Some nurse educators were unavailable for the FGI at Campus C owing to other commitments; however, they requested that the author come back as they were eager to participate. The nurse educator from Campus A may be included in this group, which was the last one for interviews, on the research supervisor’s advice after a discussion about the challenge.

Considering the limitation, the authors had in locating literature that centered on the challenges nurse educators have when developing curricula, there was little scientific evidence to support this claim. As nursing literature tended to focus more on curriculum implementation, the literature used in the discussion was predominantly about the experiences of schoolteachers.

### Recommendations

This study’s findings revealed the emotional, physical and psychological impact the curriculum development process had on the nurse educators. The recommendation is that debriefing sessions for nurse educators should be undertaken with the Gauteng Department of Health (GDoH) counsellor or psychologist. In addition, nursing education programmes at tertiary institutions should ensure that curriculum development modules also include the criteria and requirements of the CHE to ensure nurses are upskilled in developing a curriculum, because those who developed the programmes before had no exposure to these developments because the nursing programmes were not aligned to the Higher Education Qualifications Sub-Framework (HEQSF).

The authors further recommend that management at the NEIs should re-emphasise implementation of the Communication Policy Guidelines to enhance effective communication; if not in place, they should be developed and implemented. There must be monitoring of the implementation of these guidelines because it was evident from the data collected that communication was a challenge for most participants. The NEIs’ executive management should ensure that staff members have support in all projects they are tasked with, as this fosters motivation and boosts individuals’ morale. Finally, the authors recommend the undertaking of more studies regarding the experiences nurse educators in other contexts, such as in other provinces and universities, have during curriculum development and implementation thereof.

## Conclusion

The objective of this study was achieved as the challenges that nurse educators experienced were explored and described. The nurse educators revealed that they had to make provisions to meet the objectives for developing new curricula in various ways including sacrificing their own studies, using their own data and laptops as well as travelling excessively at short notice. A lack of information, a lack of support, inadequate communication and the emotional and psychological toll the curriculum development process took on nurse educators were among the difficult challenges they had to overcome.
